# A new model and precious tool to study molecular mechanisms of macrophage aging

**DOI:** 10.18632/aging.206124

**Published:** 2024-10-03

**Authors:** Rémy Smith, Kévin Bassand, Ashok Dussol, Christophe Piesse, Eric Duplus, Khadija El Hadri

**Affiliations:** 1 Sorbonne Université, CNRS UMR 8256 Biological Adaptation and Ageing (B2A), INSERM U1164, Institut de Biologie Paris Seine (IBPS), Paris 75005, France; 2 INSERM U1148, Laboratory for Vascular and Translational Sciences (LVTS), Université Sorbonne Paris Nord, Bobigny 93000, France; 3 Sorbonne Université, CNRS, Institut de Biologie Paris Seine (IBPS), Plate-forme Ingénierie des Protéines et Synthèse Peptidique, Paris 75005, France

**Keywords:** aging, macrophage, inflammation, senescence, thioredoxin-1 mimetic peptide

## Abstract

The accumulation of senescent cells, characterized by a senescence-associated secretory phenotype (SASP), contributes to chronic inflammation and age-related diseases (ARD). During aging, macrophages can adopt a senescent-like phenotype and an altered function, which promotes senescent cell accumulation. In the context of aging and ARD, controlling the resolution of the inflammatory response and preventing chronic inflammation, especially by targeting macrophages, must be a priority. Aging being a dynamic process, we developed a model of *in vitro* murine peritoneal macrophage aging. Our results show that macrophages cultured for 7 or 14 days exhibit a senescence-like phenotype: proliferation decrease, the levels of cyclin-dependent kinase inhibitors p16^INK4A^ and p21^CIP1^ and of pro-inflammatory SASP components (MCP-1, IL-6, IL-1β, TNF-α, and MMP-9) increase, phagocytosis capacity decline and glycolytic activity is induced. In our model, chronic treatment with CB3, a thioredoxin-1 mimetic anti-inflammatory peptide, completely prevents p21^CIP1^ increase and enables day 14 macrophages to maintain proliferative activity.

We describe a new model of macrophage aging with a senescence-like phenotype associated with inflammatory, metabolic and functional perturbations. This model is a valuable tool for characterizing macrophage aging mechanisms and developing innovative strategies with promising therapeutical purpose in limiting inflammaging and ARD.

## INTRODUCTION

Aging is a biological phenomenon that can impact many organs and physiological functions and represents the main risk factor for age-related diseases (ARD), such as neurodegenerative, cardiovascular, bone and muscular diseases [1]. A chronic, low-grade, systemic and sterile inflammation occurs with aging, in the absence of any infection, known as “inflammaging” [2]. Moreover, this phenomenon is crucially involved in the aetiology and progression of ARD, and may finally lead to organ failure and death [3]. An acute inflammatory response involves the recruitment of inflammatory cells from the blood, together with tissue-resident immune cells, essentially monocytes and macrophages. Importantly, the duration and the intensity of the inflammatory response is controlled by the secretion of factors like cytokines and chemokines that attract innate and adaptive immune cells to the site of damage. A successful inflammatory response is followed by a resolution phase, where the inflammatory response is gradually shut down, a critical step for restoring tissular homeostasis [4]. A major characteristic of inflammaging is the chronic activation of the innate immune system, in which the macrophage has a central role modulating the levels of pro- and anti-inflammatory factors. Other important processes are involved in inflammaging such as oxidative stress, defined as the imbalance between the production of intracellular reactive oxygen species (ROS) and cellular antioxidant capacity. It also represents a strong modulator of inflammation and is proposed as oxi-inflammaging, an oxidation-inflammatory theory of aging [5].

Cellular senescence has been suggested as an aging hallmark that is believed to contribute to inflammaging and chronic diseases. Cellular senescence is a heterogeneous process guided by genetic, epigenetic and environmental factors, characterizing many types of somatic cells [6]. The cellular senescence program is a paradigm of antagonistic pleiotropy; it acts beneficially against damage and oncogenic signaling in young people but turns deleterious with increasing age leading to age-related deterioration of the tissue’s response, function and regenerative capacity. Senescent cells exhibit a combination of biomarkers, as each marker in isolation is prone to false positives. These include high expression of the tumor suppressor molecules p16^INK4A^ and p21^CIP1^; lysosomal hydrolase activity detectable at pH 6.0, known as senescence-associated β-galactosidase (SA-β-gal) [7]; and expression of specific senescence-associated secretory phenotype (SASP), mainly characterized by the production of proinflammatory and matrix-degrading molecules [8, 9]. Senescent cells recruit cells of the immune system to organize their removal, but with increasing age, it becomes sluggish or otherwise impaired [10, 11]. The immune system exhibits remarkable changes during aging called “immunosenescence”, a multifactorial phenomenon that affects both innate and adaptive immunity and play a critical role in most chronic diseases in the elderly [12–14]. Immunosenescence is implicated in the accumulation of senescent cells in numerous tissues in aged animals, that is believed to contribute to aging, age-related dysfunction and chronic diseases [15–17]. In addition, senescent cells recruit macrophages and can induce them to undergo a senescent-like phenotype and the interaction between macrophages and senescent cells would have an impact on the effector functions of macrophages during inflammaging [18–22].

The constant immune challenges throughout the lifetime may lead to a higher basal activation state of the innate immune system, particularly monocytes and macrophages, and play a crucial role in inducing inflammaging [23–25]. In addition, damaged macromolecules, organelles, and cell debris can induce innate immunity through the NF-κB pathway and the induction of the canonical NLRP3 inflammasome [26]. Recent studies showed that the resolution of inflammation was defective in older people leading to a prolonged inflammatory response [27]. In addition, it has been reported that macrophages can become p16^INK4A^/SA-β-gal-positive cells on contact with senescent cells in the peritoneum of aging mice [28], which should be considered as a possible contributor to aging and its associated pathologies [21, 29]. In addition, the clearance of these senescent macrophages helps to improve these pathologies [30]. Indeed, aging-dependent tissue microenvironment results in a plethora of phenotypic, metabolic and functional alterations in macrophages [25]. Elevated pro-inflammatory mediators in aging reduces the capacity of peritoneal and bone marrow-derived macrophages to ingest and clear bacteria further perpetuating an inflammatory phenotype [31]. In splenic and thioglycolate-elicited peritoneal macrophages, reduced Toll-like Receptor (TLR) expression leads to reduced pro-inflammatory cytokine production in response to TLR stimuli in old as compared to young macrophages [32, 33]. It has also been reported that macrophages collected from old mice showed reduced phagocytic capacity as compared to macrophages obtained from young mice [34]. On the other hand, the metabolic state could be the cause of the switch to a proinflammatory phenotype of macrophages in aged tissues. Indeed, cellular stressors encountered with age, such as telomere stress, cause mitochondrial metabolic dysfunction, leading to increased ROS production, NLRP3 inflammasome activation and IL-1β release [35]. In order to limit inflammaging, the development of new therapeutic strategies targeting phenotypic and functional alterations in macrophages would be a major challenge.

Thioredoxin-1 (Trx-1) system represents an important antioxidant and anti-inflammatory system involved in a number of clinical conditions [36]. Various studies have reported a protective effect of Trx-1 particularly in the context of cardiovascular diseases. Trx-1 can improve endothelial function and is able to rescue endothelial cells from age-induced disorders [37]. *In vitro*, human recombinant Trx-1 downregulates the expression of a number of inflammatory genes such as IL-1β, TNF-α, and IL-6 in human macrophages [38]. Moreover, Trx-1 induces anti-inflammatory M2 macrophage polarization through downregulation of p16^INK4A^ and reduces proinflammatory M1 polarization [39]. In this context, Trx-1 mechanism of action involves Akt-1 pathway [40], and inhibits NLRP3 inflammasome [41]. In addition, Couchie et al. have shown that the circulating level of Trx-1 decreased in healthy old subjects (>65 years) compared to young individuals (<40 years) [40]. The decrease of Trx-1 with age could contribute to the loss of anti-inflammatory protection and consequently, at least in part, to the occurrence of oxidative stress, inflammaging, and pathologies development in elderly. We have recently developed a new therapeutic strategy based on a Trx-1 mimetic peptide named CB3. We previously demonstrated that CB3 exerts anti-oxidative and anti-inflammatory effects in murine peritoneal macrophages *in vitro* and in a mouse model of atherosclerosis [42]. However, the beneficial effects of CB3 in the context of aging remain to be demonstrated.

In the context of aging and ARD, controlling the resolution of the inflammatory response and preventing chronic inflammation, especially by targeting macrophages, must be a priority. Aging being a dynamic process, we first developed a new model of macrophage aging *in vitro*. We found that murine peritoneal macrophages do indeed gradually undergo a senescence-like phenotype when cultured for 14 days. *In vitro* aged macrophages presented proliferation decrease, pro-inflammatory and senescence markers increase, phagocytosis capacity decline and glycolytic induction. This model provides a precious tool for testing innovative therapeutical strategies targeting macrophages to limit inflammaging and ARD.

## RESULTS

During aging, macrophages were shown to share some phenotypic similarities with senescent cells [19–22]. These modifications lead to functional alterations contributing to inflammaging [25]. Although macrophages are unlikely to be the only source of inflammaging, therapeutic strategies targeting these cells have shown beneficial effects in the context of age-related diseases and the development of models for studying macrophages in that case is essential. Given the difficulty of working with elderly mice to isolate aged macrophages, we propose to develop a new study model analyzing molecular and functional markers of senescence and aging of murine peritoneal macrophages derived from young mice and cultured over a long term from 2 to 14 days after seeding.

**Figure 1:**
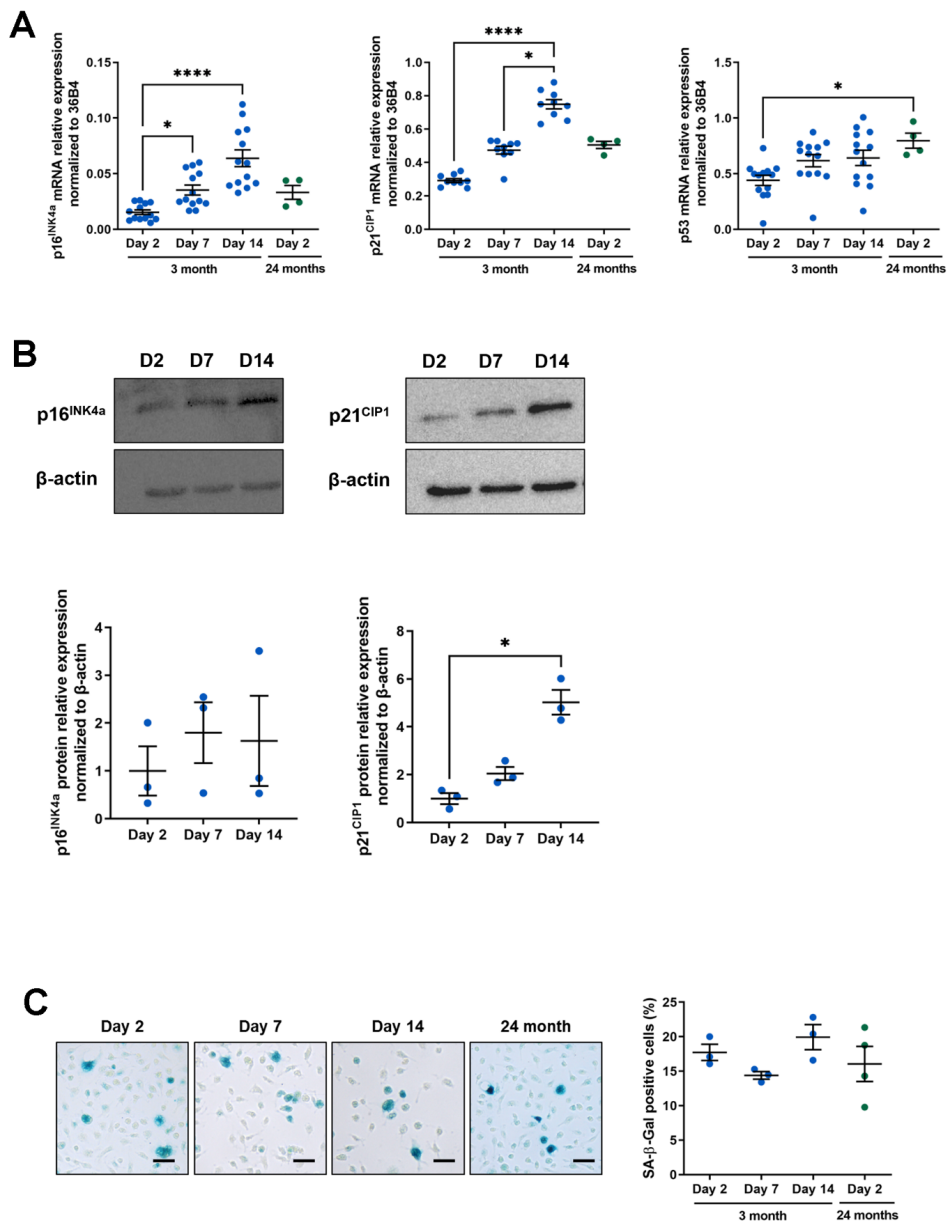
**Macrophage aging *in vitro* and senescence-associated phenotype.** Murine peritoneal macrophages from young mice (3 months) were cultured during 2, 7 or 14 days *in vitro*. All results are compared to murine peritoneal macrophages from 24-month-old mice cultured for 2 days. (**A**) RT-qPCR analysis for *p16^INK4a^*, *p21^CIP1^* and *p53* transcripts normalized to *36B4* (n=13). (**B**) Immunoblots for p16^INK4^ and p21^CIP1^ at day 2, 7 and 14 (D2, D7 and D14 respectively) with quantification by densitometric analysis (n=3-4). (**C**) Left panel: SA-b-Gal staining for 19 hours on fixed macrophages (scale bar 20 μm). Right panel: quantification of the percentage of SA-β-gal positive cells (n=3). Error bars represent the mean ± SEM. p-values were obtained comparing groups overtime using a non-parametric one-way ANOVA analysis (Kruskal-Wallis analysis followed by Dunnett’s multiple comparison test) (*p<0.05; ****p<0.0001).

Senescence is characterized in particular by increased expression of p16^INK4A^ and p21^CIP1^, two cyclin-dependent kinase inhibitors (CDKI) involved in the two major tumor suppressor pathways enabling permanent and irreversible cell cycle arrest [43]. We analyzed the *p16^INK4A^* and *p21^CIP1^* mRNA (Figure 1A) which are both significantly induced at days 7 and 14 in a time-dependent manner compared to day 2 (for *p16^INK4A^*: 2.29 and 4.15 fold induction at day 7 and 14 respectively compared to day 2; for *p21^CIP1^*: 1.62 and 2.56 fold induction at day 7 and 14 respectively compared to day 2). In contrast, mRNA encoding the tumor suppressor p53, known to be induced in certain senescent cells, is not increased. *p16^INK4A^* and *p21^CIP1^* mRNA were induced at a level relatively similar to those obtained in 2 days cultured peritoneal macrophages from 24-month-old mice (Figure 1A) so we further analyzed the p16^INK4A^ and p21^CIP1^ proteins by immunoblotting (Figure 1B). There was a tendency for p16^INK4A^ to increase with a 1.6 to 1.8 fold induction at day 7 or 14 compared to day 2 but the most significant induction was seen with p21^CIP1^ (2.05 and 5.02 fold induction at day 7 and 14 respectively compared to day 2). Because of this increase in p21, we also analyzed the p53 protein by immunoblot, despite the fact that its mRNA was not modified. p53 is indeed enhanced at D14 compared to D2 (2.71 fold induction) (see Supplementary Figure 1). SA-β-gal activity is also a feature of senescent cells and has been previously described to be induced in resident macrophages from aged mice [21]. This biomarker was also quantified both in our model and in peritoneal macrophages isolated from 24-month-old mice as previously described (Figure 1C). Surprisingly, the percentage of SA-β-gal positive cells remains unchanged over time (17.73 ± 1.18% at day 2; 14.39 ± 0.54% at day 7; 19.94 ± 1.81% at day 14) and is very similar from day 2 to 24-month-old-mice derived macrophages (16.05 ± 2.54%) suggesting that SA-β-gal activity is relatively high in cultured macrophages as early as day 2.

**Figure 2:**
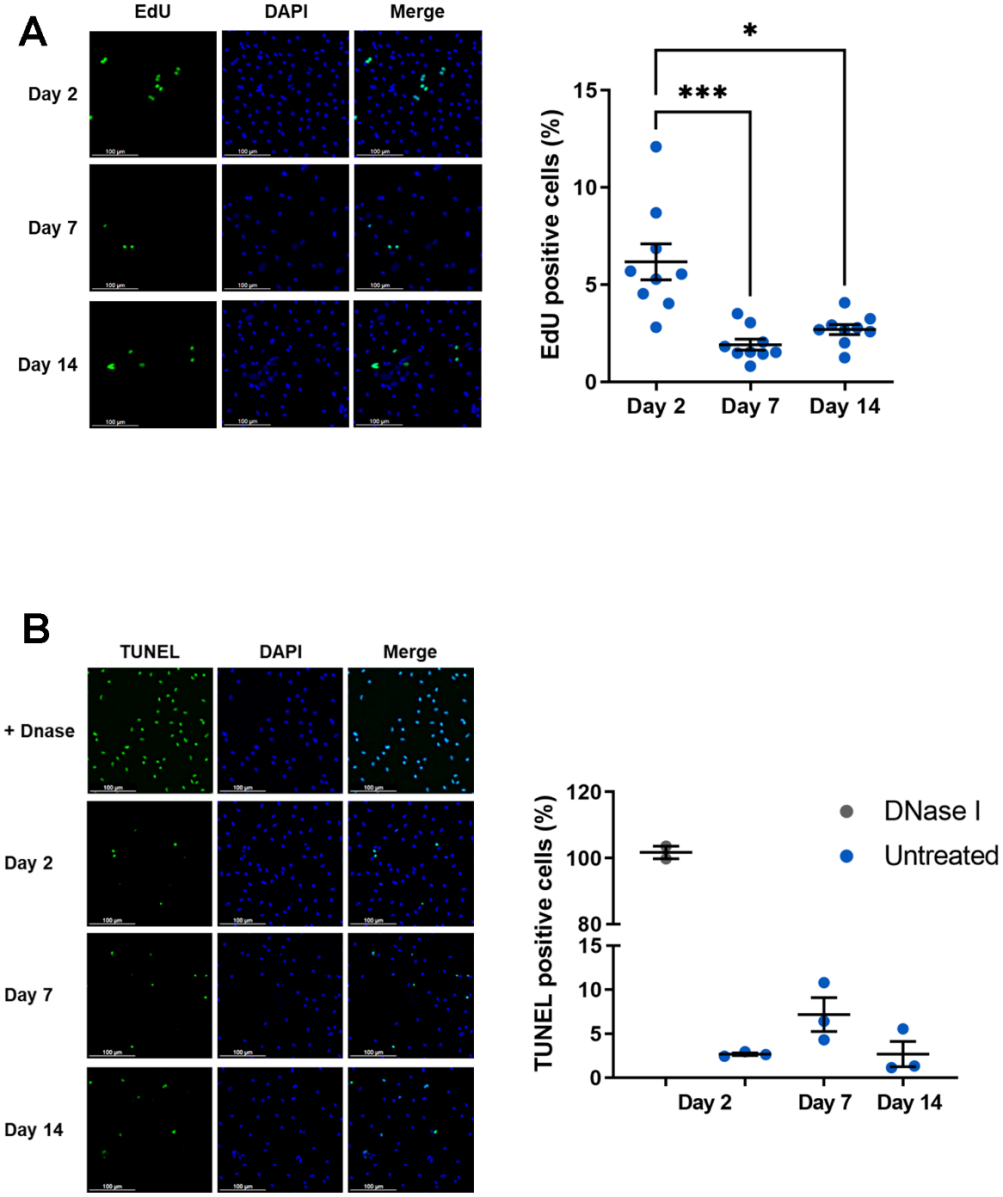
**Proliferation and apoptosis of aged macrophages *in vitro*.** Murine peritoneal macrophages from young mice (3 months) were cultured during 2, 7 or 14 days *in vitro*. (**A**) Left panel: EdU incorporation assay (scale bar 100 μm). Right panel: quantification of EdU-positive cells. EdU was added for 24 h before analysis (n=9). (**B**) Left panel: DNA fragmentation detected by TUNEL. Apoptotic cells were visualized as green, and the nuclei as blue (scale bar 100 μm). Right panel: quantification of TUNEL positive cells (n=3). Error bars represent the mean ± SEM. p-values were obtained comparing groups overtime using a non-parametric one-way ANOVA analysis (Kruskal-Wallis analysis followed by Dunnett’s multiple comparison test; *p<0.05; ***p<0.001).

As senescence-associated CDKI were induced in our model, we wondered whether macrophage proliferation is affected in these conditions. To evaluate a potential cell cycle arrest in our model of macrophage aging, cell proliferation was assessed using EdU incorporation (Figure 2A). At day 2, nearly 6.17% ± 0.92 of EdU-positive cells was detected and it was reduced by 2 to 3-fold at days 7 and 14 (1.91 % ± 0.28 and 2.68 % ± 0.26 respectively). Macrophages in culture thus have a relatively limited capacity to proliferate. However, this is affected in aged macrophages and this effect is associated with CDKI expression, particularly p21^CIP1^. Cell cycle regulators involved in senescence are known to influence proliferation but also apoptotic death which are intimately linked. In particular, p21^CIP1^ has been shown to inhibit apoptosis that could be modified in our model [44]. We further analyzed apoptosis by quantifying fragmented DNA using a TUNEL assay (Figure 2B). While DNA fragmentation by DNaseI leads to the labeling of all cells, only a small percentage of macrophages are labeled in the absence of genotoxic agents, regardless of the number of days in culture (2.7 ± 0.15 % of positive cells at day 2 vs. 7.17 ± 1.91% at day 7 vs. 2.7 ± 1.43% at day 14). Thus, apoptosis is relatively low from day 2 and cannot be further reduced as macrophages age.

**Figure 3:**
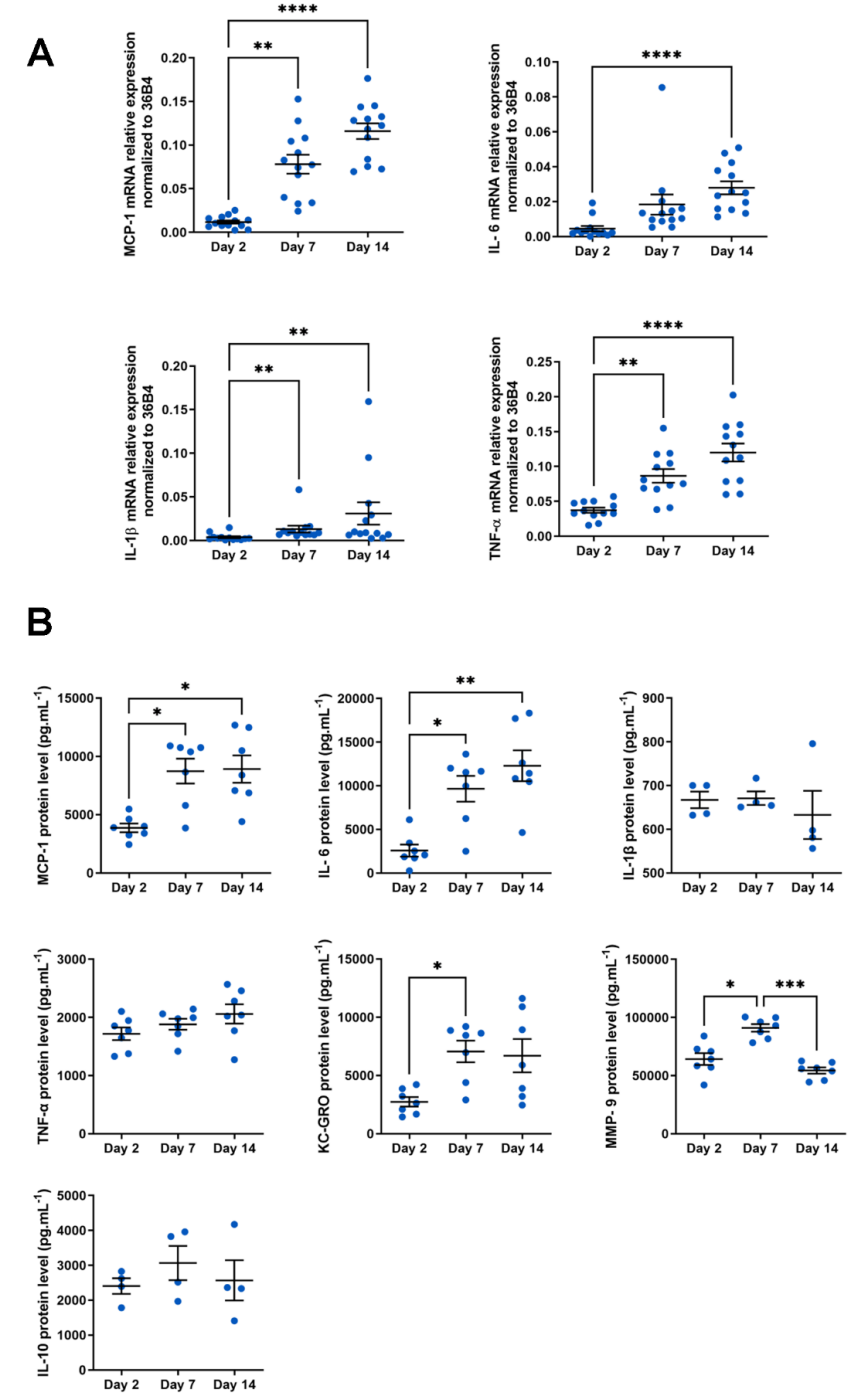
**SASP analysis in aged macrophages *in vitro*.** Murine peritoneal macrophages from young mice (3 months) were cultured during 2, 7 or 14 days *in vitro*. (**A**) RT-qPCR analysis for SASP markers MCP-1, IL-6, TNF-a and IL-1b transcripts normalized to 36B4 (n=13). (**B**) MCP-1, IL-1β, IL-6, TNF-α, KC-GRO, IL-10 and MMP-9 protein levels assessed by MSD multiplex on conditioned media harvested 24h after serum deprivation (n=4-7). Error bars represent the mean ± SEM. p-values were obtained comparing groups overtime using a non-parametric one-way ANOVA analysis (Kruskal-Wallis analysis followed by Dunnett’s multiple comparison test; *p<0.05; **p<0.01; ***p<0.001; ****p<0.0001).

Finally, senescent cells are also characterized by a pro-inflammatory secretome called SASP, composed of chemokines, cytokines, matrix metalloproteases and other components [16]. Macrophages are known to secrete numerous pro- or anti-inflammatory cytokines, depending on their phenotype and microenvironment. In the context of aging, macrophages adopt a pro-inflammatory SASP-like secretome [45]. In our model, we analyzed typical components of SASP produced by macrophages at the mRNA level using RT-qPCR (Figure 3A). The four transcripts tested (*MCP-1*, *IL-6*, *IL-1β* and *TNF-α*) were all induced in aged macrophages. Of these, *MCP-1* and *IL-1β* mRNA expression were highly increased (at day 14, 9.8 fold induction for *MCP-1 and* 8.4 fold induction for *IL-1β* vs. day 2). We also compared the expression level of mRNAs encoding SASP components of our macrophage model with those from macrophages derived from 24-month-old mice. Surprisingly, the latter are relatively low (see Supplementary Figure 2). These results suggest a high phenotypic plasticity of aged macrophages isolated from their initial context while long-term culture of macrophages from young mice maintains inflammation markers.

Some cytokines, such as IL-1β or TNF-α are known to be differentially controlled at transcriptional and translational levels [46, 47]. Protein levels of SASP molecules secreted by macrophages were therefore analyzed using a multiplex assay (Figure 3B). IL-1β and TNF-α, unlike mRNA level, protein expression does not vary in our model. As observed for their mRNAs, MCP-1 and IL-6 expression are also increased at the protein level in aged macrophages (8913 ± 1171 and 3871 ± 371 pg.mL^-1^ at day 14 vs. day 2 respectively for MCP-1; 13568 ± 1441 and 2953 ± 679 pg.mL^-1^ at day 14 vs. day 2 respectively for IL-6). We also evaluate protein level of KC-GRO (the IL-8 related protein in rodent), usually and consistently expressed by senescent cells (Figure 3B). KC-GRO is induced in macrophages with a maximal induction as soon as day 7 (7059 ± 933 at day 7 vs. 2736 ± 405 pg.mL^-1^ at day 2). Noteworthy is the significant induction of MCP-1, IL-6 and KC-GRO secretion, which are important and pleiotropic pro-inflammatory components of SASP [48].

To further characterize our *in vitro* macrophage aging model, we looked at the matrix metalloproteinase MMP-9 involved in cytokine and chemokine activation in age-related pathologies [49] (Figure 3B). MMP-9 is transiently induced at day 7 (91056 ± 3302 at day 7 vs. 64221 ± 5086 pg.mL^-1^ at day 2). In contrast, IL-10, a cytokine with anti-inflammatory properties, is not altered in our model regardless of the macrophage culture stage. Thus, in our macrophage aging model, we have shown an induction of molecules specifically and strongly involved in inflammation and age-related pathologies (MCP-1, IL-6, KC-GRO and MMP-9), while an anti-inflammatory cytokine such as IL-10 is not altered. These results make this experimental device ideal for analyzing the pro-inflammatory SASP produced by macrophages in the context of age.

**Figure 4:**
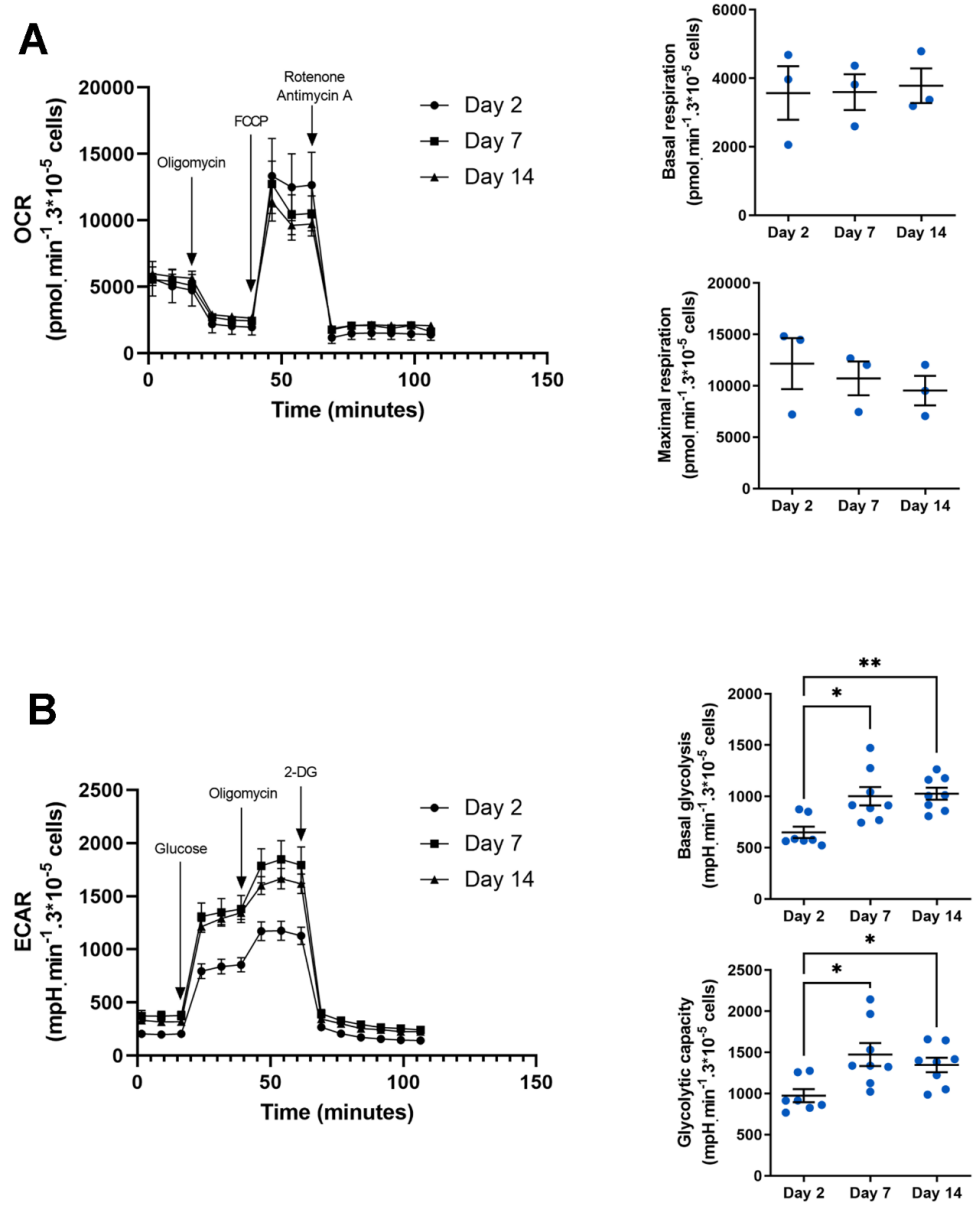
**Metabolic phenotype in aged macrophages *in vitro*.** (**A**) Seahorse analysis of oxygen consumption rate (OCR), basal and maximal respiration. Results are normalized by cell count using DAPI staining (n=3). (**B**) Seahorse analysis of extracellular acidification rate (ECAR), basal glycolysis and glycolytic capacity. Results are normalized by cell count using DAPI staining (n=8). Error bars represent the mean ± SEM. p-values were obtained comparing groups overtime using a non-parametric one-way ANOVA analysis (Kruskal-Wallis analysis followed by Dunnett’s multiple comparison test; *p<0.05; **p<0.01).

The pro- or anti-inflammatory phenotype of macrophages is highly dependent on intracellular metabolism. Indeed, M1 macrophages tend to have a glycolytic metabolism, while M2 macrophages require a more oxidative metabolism [50]. These metabolic adaptations are necessary to match macrophage function to changes in its microenvironment. For example, increasing glycolytic flux enables an increase in the pentose phosphate pathway needed to produce NADPH used to generate ROS [51]. To assess the metabolic profile of macrophages in our model, we measured oxygen consumption rate (OCR) (Figure 4A) and extracellular acidification rate (ECAR) (Figure 4B) using SeaHorse technology. These parameters enabled us to deduce basal respiration and glycolysis, as well as maximal glycolytic and respiratory capacity. The assay showed that OCR profile but also basal respiration and maximal respiration are not different whatever the day of culture (Figure 4A) whereas ECAR profile is increased at days 7 and 14 compared to day 2 (Figure 4B). These differences show an increase in both basal glycolytic activity (1026 ± 57 and 649 ± 55 mpH/min/3.10^5^ cells at day 14 vs. day 2 respectively) and glycolytic capacity (1346 ± 87 and 974 ± 78 mpH/min/3.10^5^ cells at day 14 vs. day 2 respectively) suggesting that glucose consumption and glycolytic flux are enhanced to supply the metabolites required for the macrophage to acquire a pro-inflammatory phenotype.

**Figure 5:**
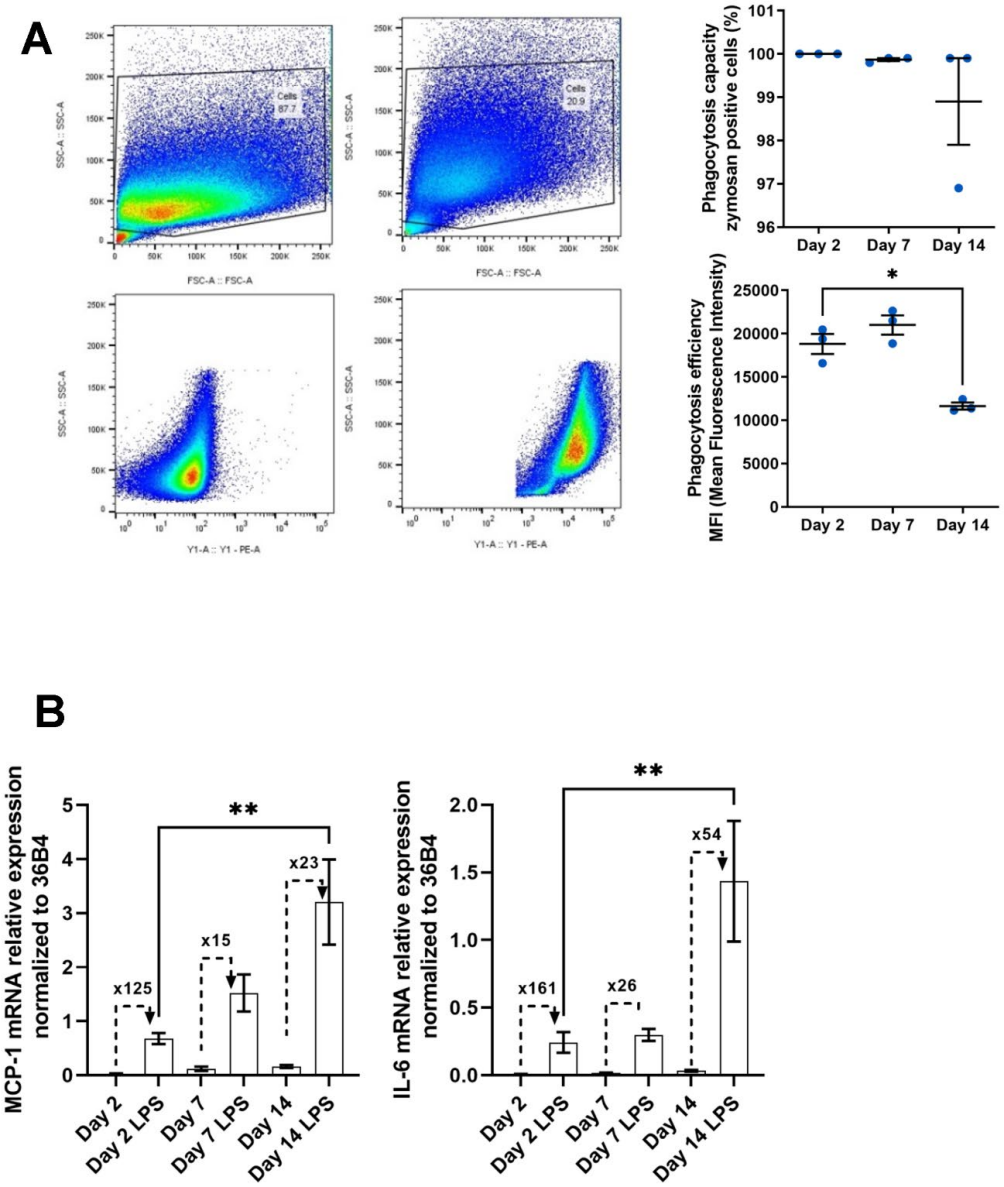
**Functional alterations in aged macrophages *in vitro*.** (**A**) Gating strategy for flow cytometry analysis of phagocytosis after 3h of pHrodo Red zymosan treatment. Right upper panel: Quantification of the number of phagocytic macrophages (% of positive cells). Right below panel: Quantification of the mean fluorescence intensity (MFI) representing phagocytosis capacity (n=3). (**B**) Macrophages are incubated with LPS (10 ng/mL) for 24h. Quantification of the pro-inflammatory response to LPS by RT-qPCR by measuring the relative expression of MCP-1 and IL-6 mRNAs. Fold induction is expressed between the control and the LPS (n=7). Error bars represent the mean±SEM. p-values were obtained comparing groups overtime using a non-parametric one-way ANOVA analysis (Kruskal-Wallis analysis followed by Dunnett’s multiple comparison test; *p<0.05; **p<0.01).

In addition to the fact that macrophages increase their production of senescence markers and pro-inflammatory molecules with some metabolic modifications, these cells also undergo functional alterations with age. One of the main functions of macrophages is to eliminate pathogens and cellular debris accumulated during aging via phagocytosis [52, 53]. This function has been extensively studied showing its impairment in the context of aging. This alteration leads to chronic inflammation due to its unresolved nature [34, 54]. We further analyzed phagocytic activity in our model of peritoneal macrophages aging *in vitro* using a phagocytosis assay with Zymosan substrate (Figure 5A) [55]. Phagocytosis activity was quantified by flow cytometry. After verifying that all cells were capable of phagocytosis under our experimental conditions (Figure 5A, upper panel), the fluorescence intensity of phagocytosed Zymosan particles was measured (Figure 5A, bottom panel). We observed a decrease in phagocytosis of nearly 40% on day 14 compared to days 2 and 7 (11645 ± 396 and 18820 ± 1156 MFI at day 14 and day 2 respectively).

Among phagocytes such as monocytes/macrophages, but also dendritic cells, the age-related reduction in phagocytic activity has been associated with a decrease in the expression of some TLR [33]. These are involved in the recognition of pathogens and pro-inflammatory molecules such as LPS, leading to increased expression and secretion of inflammatory cytokines. As a result, the immune system is often less reactive to the presence of exogenous pathogens. Despite the decrease in these receptors, the expression of these cytokines is usually deregulated independently of the microenvironment [54]. In this context, we wanted to test the ability of our macrophage model to respond to LPS (Figure 5B). Following 24hs-LPS induction of our cells at various stages of culture, we analyzed the expression of *MCP-1* and *IL-6* transcripts by RT-qPCR, given that these two transcripts and their associated proteins are induced at a basal level in our aging model (Figure 3A, 3B). We observed an increase in the level of these molecules in response to LPS (3.21 ± 0.79 and 0.68 ± 0.10 at day 14 and day 2 respectively for *MCP-1*; 1.43 ± 0.44 and 0.24 ± 0.07 at day 14 and day 2 respectively for *IL-6*) but the fold induction observed with LPS compared to control condition was decreased at day 14 as basal level is higher compared to day 2 (23 and 125 fold induction at day 14 compared to day 2 for *MCP-1*; 54 and 161 fold induction at day 14 compared to day 2 for *IL-6*). Macrophages thus show deregulation of basal expression of pro-inflammatory molecules and a reduction in the LPS-induced immune response.

Characterization of this new macrophage aging model has revealed phenotypic alterations (proliferation, CDKI, metabolism and pro-inflammatory secretory phenotype) as well as functional alterations (phagocytosis and immune response). Given these alterations, we wanted to determine whether these aged macrophages could be sensitive to the action of senolytics. To do this, we treated murine peritoneal macrophages aged 2, 7 and 14 days with 250 nM dasatinib and 15 or 30 μM quercetin for 24 hours (See Supplementary Figure 3). Surprisingly, aged macrophages are resistant to the effect of senolytics, as has already been shown elsewhere [56]. These results suggest that these macrophages acquire a non-classical senescent phenotype known as senescent-like. This model provides an easy-to-use tool for testing therapeutic molecules aimed at limiting macrophage aging and associated inflammaging. In this context, we are interested in Trx-1 oxydo-reductase, an important enzyme which has been shown to be involved in the control of senescence and aging in endothelial cells and fibroblasts [57, 58]. We focus on CB3, a peptide derived from the catalytic site of the Trx-1 enzyme, as we have previously shown that CB3 possesses interesting anti-inflammatory properties in peritoneal macrophages derived from young mice and treated with LPS. In particular, CB3 reduces the secretion of MCP-1, IL-1β, IL-6 and TNF-α [42]. Given its properties, this peptide offers undeniable preventive and therapeutic prospects in the field of age-related pathologies. However, the effects of this peptide have never been characterized in the context of aging.

**Figure 6:**
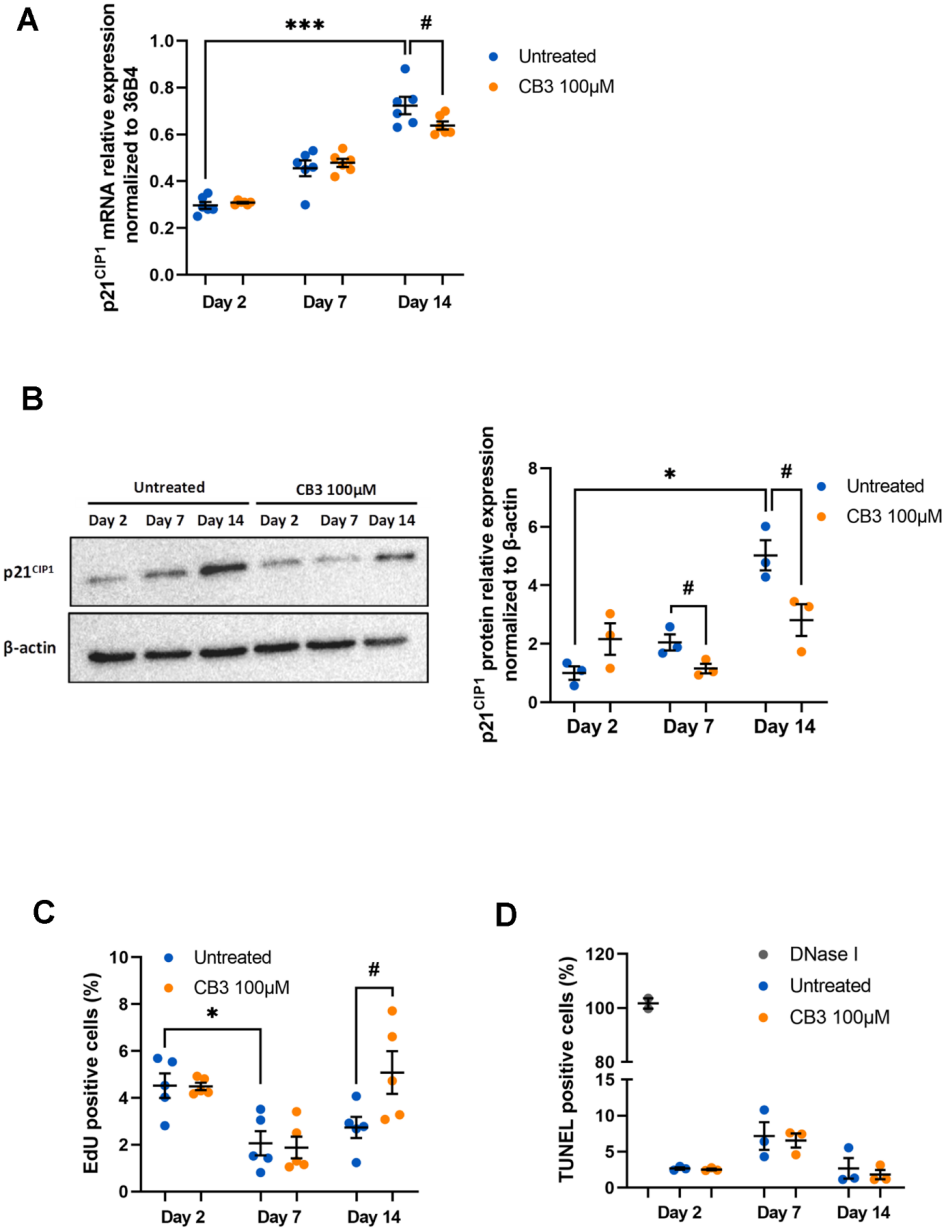
**CB3 effects on proliferation and apoptosis in aged macrophages *in vitro*.** Macrophages are chronically treated with 100uM CB3 for up to 14 days. (**A**) RT-qPCR analysis for p21^CIP1^ transcripts normalized to 36B4 (n=7). (**B**) Immunoblot and p21^CIP1^ quantification by densitometric analysis (n=3). (**C**) Quantification of EdU-positive cells. EdU was added for 24 h before analysis. (**D**) Quantification of TUNEL positive cells (n=3). Error bars represent the mean ± SEM. p-values were obtained comparing groups overtime using a non-parametric one-way ANOVA analysis (Kruskal-Wallis analysis followed by Dunnett’s multiple comparison test; *p<0.05; ***p<0.001) or using a non-parametric t-test (Mann-Whitney) to analyze the significance between groups treated or not treated with CB3 (#*p*<0.05).

To test this, we treated peritoneal macrophages chronically with 100μM CB3, an optimal concentration previously tested on young macrophages in our previous work [42]. First, we analyzed *p21^CIP1^* mRNA and protein, both of which are increased in aged macrophages in our model (Figure 1A, 1B). Here we show that CB3 induces a small reduction in *p21^CIP1^* mRNA at day 14 (0.64 ± 0.02 vs. 0.72 ± 0.04 at day 14 with or without CB3 respectively) (Figure 6A), while the protein is more strongly decreased as early as day 7 (1.15 ± 0.16 and 2.05± 0.27 at day 7 with or without CB3 respectively; 2.81 ± 0.54 and 5.03± 0.51 at day 14 with or without CB3 respectively) (Figure 6B). This result suggests that p21^CIP1^ could be differentially regulated at the transcriptional and translational level as it was already described before [59]. CB3-induced decrease in p21^CIP1^ is accompanied by restoration of macrophage proliferative activity to baseline at day 14 (5.08 ± 0.91 and 2.74 ± 0.45 at day 14 with or without CB3 respectively) (Figure 6C) with no change in apoptotic activity (Figure 6D). In contrast, regulation of p21 by CB3 is not accompanied by changes in p53 (See Supplementary Figure 1).

**Figure 7:**
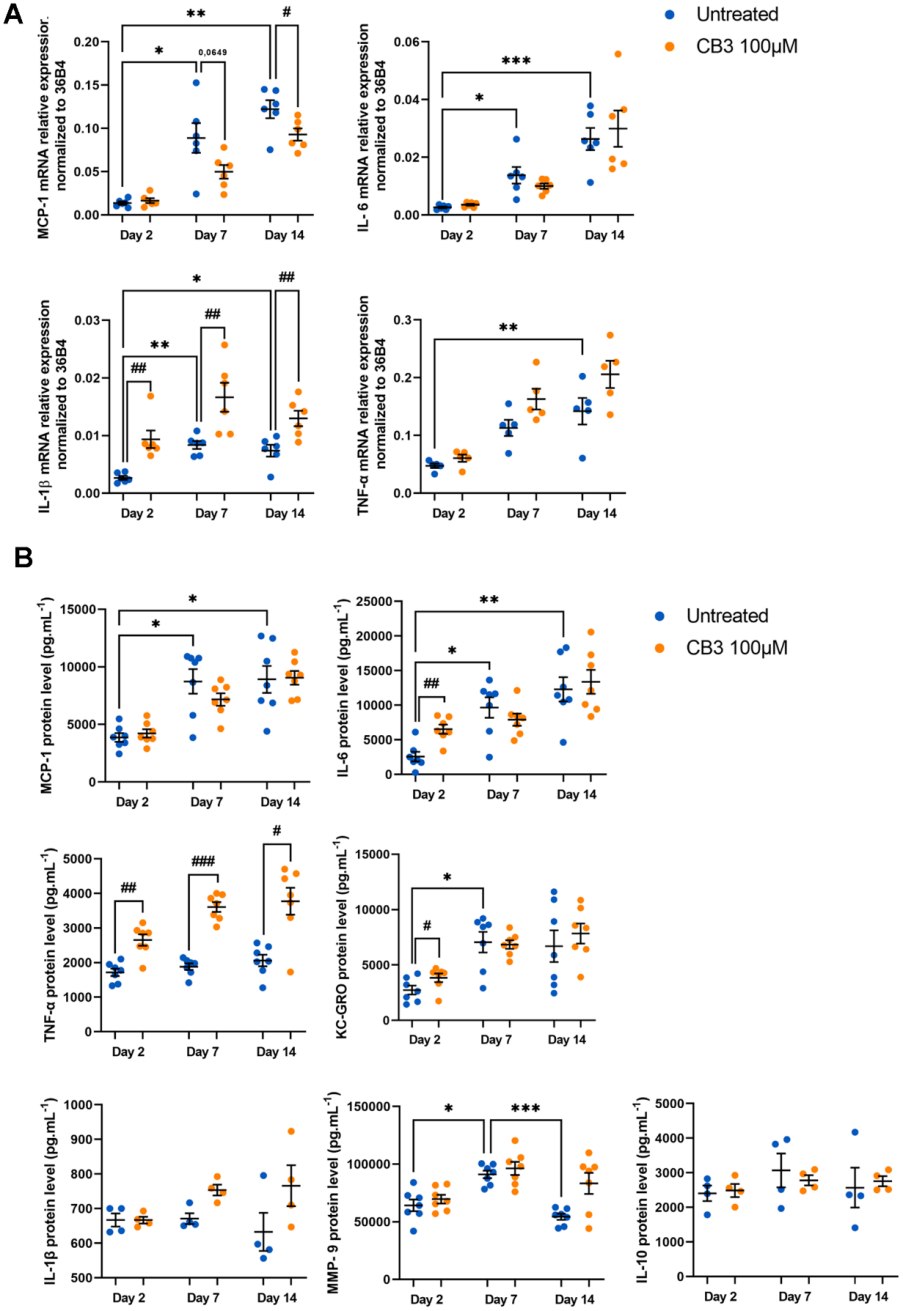
**CB3 effects on inflammatory phenotype in aged macrophages *in vitro*.** Macrophages are chronically treated with 100uM CB3 for up to 14 days. (**A**) RT-qPCR analysis for SASP markers MCP-1, IL-6, IL-1b and TNF-a transcripts normalized to 36B4 (n=7). (**B**) MCP-1, IL-1β, IL-6, TNF-α, KC-GRO, IL-10 and MMP-9 protein levels assessed by MSD multiplex on conditioned media harvested 24h after serum derivation (n=6). Error bars represent the mean ± SEM. p-values were obtained comparing groups overtime using a non-parametric one-way ANOVA analysis (Kruskal-Wallis analysis followed by Dunnett’s multiple comparison test; *p<0.05; **p<0.01; ***p<0.001) or using a non-parametric t-test (Mann-Whitney) to analyze the significance between groups treated or not treated with CB3 (#*p*<0.05; ##*p*<0,01, ###*p*<0.001).

Regarding the anti-inflammatory effects of CB3, we analyzed them on SASP components at mRNA level and also at protein level by Multiplex analysis of extracellular media as previously described (Figure 7A, 7B). We were able to demonstrate a CB3-induced decrease in *MCP-1* mRNA levels at days 7 and 14 (0.05 ± 0.007 and 0.09 ± 0.02 at day 7 with or without CB3 respectively; 0.09 ± 0.007 and 0.12 ± 0.01 at day 14 with or without CB3 respectively) (Figure 7A). Surprisingly, *IL-1β* mRNA is increased by CB3 at all stages of macrophage culture (0.01 ± 0.001 and 0.008 ± 0.001 at day 14 with or without CB3 respectively). Most compounds are not controlled by CB3 except for IL-6 and KC-GRO which are significantly induced by the peptide at day 2 (2.21 ± 0.22 and 1.41 ± 0.37 fold induction respectively) and TNF-α at all stages (1.54 ± 0.25, 1.92 ± 0.20 and 1.83 ± 0.50 fold induction at days 2, 7 and 14 respectively) (Figure 7B). Thus, while CB3 can modulate a senescence marker such as p21^CIP1^, this peptide has no anti-inflammatory properties in our *in vitro* aged macrophages. Finally, our results show no effect of CB3 either on glycolytic activity or on phagocytosis activity, two parameters clearly altered in our model (see Supplementary Figures 4, 5). These results are consistent with the absence of any effect of CB3 on the inflammatory phenotype of these cells.

In conclusion, we have developed and characterized an *in vitro* aging model of murine peritoneal macrophages. In this model, we observed an increase in the expression of a CDKI p21^CIP1^ and components characteristic of SASP (MCP-1, IL-6...), an increase in glycolytic activity as well as functional deregulations such as a decrease in phagocytosis and the response to LPS stimulation. This model may thus enable the development of new therapeutic strategies aimed at reducing macrophage aging. On the other hand, CB3, whose anti-inflammatory properties have been well characterized in young macrophages induced by LPS, does not appear to possess the same effects in the context of age.

## DISCUSSION

Age-related senescence of the immune system affects both innate and adaptive immune cells, leading to multiple alterations in organs. The innate immune system, including monocytes and macrophages, plays a major role in systemic aging and is now a prime therapeutic target for improving the health of the elderly. With a half-life of several weeks [60], macrophages are much longer lived than the majority of innate immune cells. Macrophages therefore survive long enough to accumulate molecular, phenotypic and functional damages associated with senescence [24]. In this regard, Fei et al. demonstrate that metabolic dysregulation contributes to macrophage hypo-responsiveness to LPS in cultured bone marrow-derived macrophages from old mice compared to those from young mice [61]. In an *in vivo* model of peritoneal inflammation, macrophages accumulate with aging, and display characteristics of senescence including the p16^INK4a^ promoter activation, reduced proliferation, SA-β-gal activation, and increased mRNA expression of a subset of SASP factors [19]. Hall et al. also described an *in vivo* senescent-like subclass of p16^INK4a^/β-gal-positive macrophages [21].

We currently need to improve immune health in older adults with strategies targeting macrophages to improve elderly immune function and decrease inflammaging. The aging as senescence are dynamic multistep processes and are characterized by a continuous remodeling [43, 62]. To date, there is no *in vitro* model showing age-related alterations at both phenotypic, metabolic and functional levels and permitting characterization of molecular mechanisms of macrophage aging in a dynamic and controlled way. In this work we characterized a new and valuable *in vitro* aging model of murine peritoneal macrophages that presents a senescent-like pro-inflammatory phenotype with metabolic and functional alterations in accordance with scientific literature in this field.

The simplified model suggested by Herranz & Gil proposes three steps in senescence process: senescence initiation, early senescence, and late phases of senescence [43]. In accordance with this model, our *in vitro* aged macrophages present a “senescence initiation” with cell cycle exit characterized by a maximal inhibition of proliferation as early as day 7 of culture (Figure 2A). Associated with this phenomenon, we observe a strong significant increase in p21^CIP1^ mRNA and protein over time in our cultured macrophage model (Figure 1A, 1B). This biomarker of senescence is generally induced by DNA damage during aging, but also during cell differentiation and growth [63]. Peritoneal macrophages from p21^-/-^ mice express fewer pro-inflammatory factors and have increased phagocytic activity protecting against atherosclerosis [64]. Thus, p21^CIP1^ can be considered as an excellent marker of phenotypic and functional macrophage dysregulations in our model. In senescent cells, accumulation of p21^CIP1^ leads to chronic activation of Retinoblastoma Rb family proteins and inactivation of transcription factor E2F, resulting in irreversible cell cycle arrest and proliferation [65]. Interestingly, our results showed a decrease in peritoneal macrophage proliferation in culture on days 7 and 14 compared with day 2 (Figure 2A). However, this proliferation activity is relatively low as already described in previous works where the proliferation of these cells needs the addition of growth factors such as Macrophage-Colony Stimulating Factor (M-CSF) to the culture medium [66, 67]. This low proliferative activity in our model suggests that a majority of cells are in a post-mitotic state, and that the aging markers observed are part of this context as already described in the well-known post-mitotic neurons [68, 69]. For a second CDKI, p16^INK4A^ we have shown that mRNA levels significantly increase (2.29 and 4.15 fold induction at day 7 and 14 respectively compared to day 2) (Figure 1A) while protein tended to increase without statistical significance (1.6 to 1.8 fold induction at day 7 or 14 compared to day 2) (Figure 1B).

To explore senescent-like phenotype of our model, we also characterized a SA-β-gal activity and observed of around 15-20% of SA-β-gal positive cells in our model, without variation whatever the day of culture. Moreover, this percentage is comparable to that observed in macrophages derived from aged mice (Figure 1C). The expression of senescence markers by macrophages has been described several times in different monocyte/macrophage models. For example, the peritoneum of aged mice contains macrophages expressing p16^INK4A^ and SA-β-gal activity under the influence of surrounding senescent cells suggesting that macrophages may transiently adopt senescence-like phenotype [21]. Furthermore, irradiation of murine peritoneal macrophages leads to the expression of p16^INK4A^, p21^CIP1^ and SA-β-gal activity [70]. However, not all these markers are necessarily consensual in the study of macrophage aging. In particular, SA-β-gal is associated with lysosomal activity, which is important and necessary for phagocytic function [71]. On the other hand, the proliferation rate of our cells is relatively low (Figure 2A) and SA-β-gal activity is not considered a reliable marker of senescence in post-mitotic cells, some of which already possess this activity during their terminal differentiation phase [72]. Finally, p16 ^INK4A^ and SA-β-gal activity are markers that are regulated according to macrophage polarization state, without the latter being associated with senescence [28, 39]. These results suggest that, in our model, macrophages exhibit a new senescent-like phenotype induced by *in vitro* culture conditions rather than induced by a senescent cell environment [73, 74].

Macrophage phenotypic plasticity is highly dependent on the tissue microenvironment, and is reflected in specific gene expression profiles and metabolic signatures [50]. Here, we demonstrate an increase in glycolytic flux as well as maximum glycolytic capacities as early as 7 days of culture with no change in oxidative metabolism (Figure 4B). The predominance of glycolytic metabolism is one of the characteristics of pro-inflammatory M1 macrophages [75]. Increasing the number of intermediates in this pathway will enable the pentose phosphate pathway to produce more of the NADPH needed for ROS production and pathogen elimination [51]. In addition, these metabolic modifications allow the accumulation of TCA intermediates, such as citrate or succinate, which are involved in the increased transcription of proinflammatory genes as observed in our model (Figure 3) [76, 77]. In some models, the increase in glycolysis is accompanied by a decrease in oxidative metabolism. This is not the case in our model but it has been shown that metabolic changes, independently of the microenvironment, can differ according to the type of macrophage and species considered [78]. For example, large peritoneal macrophages activated by Zymosan particles or yeasts display a high oxidative metabolism required for oxidative burst [79].

The cell type undergoing senescence and how senescence is triggered, determines SASP composition. A recent analysis identified a core of SASP components common across different senescence inducers and in distinct cell types [80]. The components of SASP are numerous, but among the most important ones are: IL-6, -8 and -1β, TNF-α and matrix metalloproteinases (MMPs). The central signaling pathway for SASP generation is likely to be shared between different types of senescent cells, converging on the transcription factor NF-κB, a major regulator of inflammation in immune cells that also plays a critical role in the onset of the SASP [81–83]. In our *in vitro* aged macrophages, we characterized a SASP onsite through cytokines (i.e., IL-6 and IL-1β), chemokines (i.e., MCP-1 and KC-GRO) and metalloproteinase (i.e., MMP-9) production at mRNA and protein levels. The most highly induced cytokines and chemokines are IL-6 (4.6 fold induction at day 14 vs. day 2), KC-GRO (2.6 fold induction day 14 vs. day 2) and MCP-1 (2.3 fold induction day 14 vs. day 2).

IL-6 is a major SASP component, multifunctional, pleiotropic cytokine involved in regulation of immune responses, acute phase responses, hematopoiesis, and inflammation [84]. This cytokine is produced by endothelial cells, fibroblasts, monocytes, and macrophages in response to different stimuli (IL-1β and TNF-α) during systemic inflammation [85, 86]. Increased serum level of IL-6 is a characteristic of aging [87, 88]. In accordance with our results, blood monocyte-derived macrophages (BMDM) derived from aged mice produce more IL-6 than those derived from young animals [31]. In our *in vitro* aged macrophages KC-GRO secretion is highly and significantly induced and is maximal from day 7 of culture. IL-6 and KC-GRO expression is regulated through the activation of two main transcription factors: NF-κB and CCAAT/enhancer binding protein β (C/EBP-β) [89, 90]. In turn, IL-6 and KC-GRO act in an autocrine feed-forward loop to enhance the activity of C/EBP-β and NF-κB and amplify SASP signaling [89, 90].

IL-1β is a potent proinflammatory cytokine that is produced by tissue immune cells, particularly macrophages, fibroblasts and epithelial cells. It mediates many inflammatory diseases by initiating and potentiating immune and inflammatory responses [85]. In our model, *IL-1β* mRNA presented an 8.4 fold induction at day 14 vs. day 2 (Figure 3A). Nevertheless, there was no change in the level of secretion of this cytokine by macrophages over time. As IL-1β maturation is dependent on NLRP3 inflammasome activation, we hypothesize that the absence of inflammasome activators in our model would explain this result.

We also showed that MMP-9 expression is significantly and transiently induced over time at day 7 (Figure 3B). This result strengthens the relevance of our dynamic aging model in the study of mechanisms regulating certain processes that take place sequentially and transiently during macrophage aging.

Macrophage functional alterations described during aging have never been associated with a senescent-like phenotype. Our model of senescent-like macrophage is also characterized by functional alterations. Among its functions, phagocytosis plays an important role in the inflammatory phase with the elimination of pathogens, but also in the resolution of inflammation and tissue repair, through the efferocytosis of apoptotic cells and cellular debris. We show here that the ability of cultured peritoneal macrophages to phagocytose is significantly impaired on day 14 compared with days 2 and 7 (Figure 5A). This reduction in phagocytosis activity is described as a recurrent functional alteration in aged macrophages or monocytes in both mice and humans respectively [53, 54]. However, it seems that phagocytosis may be unaltered or even enhanced as it was described in some models like rat alveolar macrophages or microglia respectively [91, 92]. This disparity in results can clearly be explained by age-related changes in the microenvironment and would suggest that this functional alteration is reversible and represents a prime therapeutic target for stimulating the immune system in the elderly [93]. For a more detailed analysis of age-related macrophage functional alterations, we also showed a significant decrease in LPS-induced increase of MCP-1 and IL-6 as early as day 7 of culture (Figure 5B). Other studies have also shown a reduced response to TLR ligands in thioglycollate-elicited peritoneal macrophages from aged mice, with lower production of pro-inflammatory cytokines [32, 94]. Among the multiple causes of this age-related decline in macrophage immune response, several studies have suggested a decrease in TLR expression on the cell surface. Notably, expression of TLR-4, the receptor involved in LPS effects, is decreased in splenic and peritoneal macrophages from old mice compared with those from young mice. This decrease correlates with a reduction in LPS-induced TNF-α and IL-6 levels [32]. Other studies have shown a lack of variation in TLR-4 expression levels despite a decrease in LPS-dependent cytokine induction, suggesting a decrease in Mitogen-Activated Protein Kinase (MAPK) associated with signaling pathways downstream of TLRs [56]. Finally, other studies show an increase in the production of pro-inflammatory cytokines under the effect of TLR activation [31]. These seemingly contradictory results point to the importance of the macrophage microenvironment. In our model, the level of inflammatory markers induced by LPS is lower in aged macrophages, but the basal level of cytokines is higher than in young macrophages. These results suggest that the altered immune response is not necessarily linked to a decrease in TLR signaling pathways, but rather to a higher basal level of cytokines in the absence of any added inducer. This higher level is probably due to an environment that chronically stimulates macrophages, making them less sensitive to other exogenous inducers.

Given the key role of cellular senescence and immunosenescence in driving aging and many age-related diseases, various strategies have been attempted and developed to target senescent cells and aging immune cells [56, 95–97]. In our new and precious model of macrophage aging/senescence we tested a Trx-1 mimetic peptide, CB3, for the following reasons. We previously demonstrated that CB3 exerts anti-inflammatory effects on macrophages, promotes M2 anti-inflammatory phenotypic orientation, inhibits NF-κB pathway and exerts anti-atherogenic effects in young mice model [42]. Therefore, we tested whether CB3 can alleviate some or all *in vitro* age-dependent alterations in our model.

Our results showed that CB3 completely prevents p21^CIP1^ expression at protein level (Figure 6B) as early as day 7. Consistently and chronologically with the decline in p21^CIP1^ levels, chronic CB3 treatment at day 14 enables macrophages to maintain proliferative activity equivalent to that at day 2 (Figure 6C). In contrast, CB3 has no effect on p53 suggesting that p21 induction is not dependent on p53 (see Supplementary Figure 1). In our model, the peptide has no effect on *in vitro* age-dependent induction of pro-inflammatory SASP components expression (Figure 7B), on glycolytic activity or on altered phagocytosis activity (see Supplementary Figures 4, 5). The Trx-1 mimetic peptide, CB3 seems to act as an immunosenescence modulator by targeting “senescence initiation” allowing macrophages to reenter the cell cycle. Despite the anti-inflammatory effects of CB3 on young macrophages [40], we were unable to reproduce these effects in an aging macrophage context, suggesting that the anti-inflammatory effects of CB3 would be dependent on the context of aging.

Thus, we present here a new model that develops many alterations characteristic of macrophage aging with a senescent-like phenotype. This model may thus enable the characterization of mechanisms of macrophage aging and the development of new therapeutic approaches which are likely to limit inflammaging and associated pathologies.

## MATERIALS AND METHODS

### Animals

All procedures involving animal handling and their care were in accordance with the Sorbonne Université Guidelines for Husbandry of Laboratory Mice. Animals were housed in an environmentally-controlled animal facility for the duration of the experiment. Animals were received one week before the experimentation for acclimation. All animals had access ad libitum to food and water. Experiments were conducted on C57BL6/JRj females 12 weeks old mice purchased from Janvier Labs (Le Genest St Isle, France).

### Murine peritoneal macrophage isolation

Peritoneal macrophages were collected from 4% thioglycolate-injected 12-weeks-old C57BL6/JRj mice by peritoneal lavage with cold Phosphate Buffered Saline (PBS) and further centrifugation (520xg, 10 min, 4° C). Cell concentration was determined using a Nucleocounter (ChemoMetec, Allerod, Denmark) according to the manufacturer’s instructions. Cells were seeded at 3.10^6^ cells per well (6-well plate) for all experiments and at 3.10^5^ cells per well (24-well plate, Agilent Technologies, 102340-100, USA) for Seahorse analysis. Cells were seeded (D0) in RPMI 1640 medium with GlutaMAX™ (Thermo Fisher Scientific, 61870, USA) containing 10% (v/v) heat-inactivated fetal bovine serum (Thermo Fisher Scientific, 10500-064), 100U/mL penicillin and 100μg/mL streptomycin (Thermo Fisher Scientific, 15140122) in a humidified 5% CO_2_ incubator at 37° C. Cells were washed twice with PBS at D0 and D1 stages to remove non adherent cells. From seeding, macrophages were cultured for 2, 7 and 14 days. From D2, cells were chronically treated or not with 100 μM CB3 and this treatment was renewed every two days for D7 and D14 stages. At Day 2, Day 7 and Day 14 a 24h serum deprivation was performed in the absence or presence of CB3. Then, cells were harvested and RNA or proteins were extracted. Culture media were collected and frozen at -20° C.

### CB3 peptide synthesis and purification

Carboxamidated CB3 peptide was synthesized via Fmoc chemistry using the Liberty Blue automated microwave peptide synthesizer (CEM Corporation, USA), a Rink Amide MBHA resin (Merck Millipore, 855003, Germany), and a systematic double-coupling protocol. Fmoc-protected amino acids were purchased from Iris Biotech GMBH (Marktredwitz, Germany), solvents from Carlo Erba and all other reagents from Sigma-Aldrich (USA). The NH2 terminal of the crude peptide was acetylated with addition of 10% acetic acid. The peptide was cleaved from the resin and deprotected using standard TFA procedures with 1,2-ethanedithiol, water, and triisopropylsilane as scavengers. The peptide was purified by reverse-phase high-performance liquid chromatography (RP-HPLC) using a Phenomenex Luna C18 (2) semi-preparative column (5 μm, 250 × 10 mm) eluted at a flow rate of 5 mL/min by a 0-60% linear gradient of ACN (0.07% TFA) in 0.1% TFA/water (1% ACN/min). The homogeneity and identity of the synthetic peptide were assessed by matrix-assisted laser desorption/ionization-time of flight (MALDI-TOF) mass spectrometry (Voyager DE-PRO Applied Biosystems, Mass Spectrometry and Proteomics Platform, IBPS, UPMC, Paris, France) and analytical RP-HPLC (Apollo C18 column, 5 μm, 250 x 4.6 mm, W.R. Grace) using the above conditions with a flow rate of 0.75 ml/min.

### Senescence-associated-β-galactosidase staining

Twenty-four hours after serum starvation, cultured macrophages were fixed for 8 min with 10% Formalin (Sigma-Aldrich, HT501128). Cells were washed with PBS and then incubated in 40mM citric acid/phosphate buffer at pH 6.0 containing 5mM potassium ferrocyanide, 5mM potassium ferricyanide, 150mM NaCl, 2mM MgCl2, and 1mg/mL X-gal (Invitrogen, B1690, USA) for 19 hr at 37° C in the dark. After incubation, cells were washed with PBS and images were acquired by optical microscopy using an Olympus E-620 camera. Cells with SA-β-gal activity were quantified using ImageJ software (NIH, v1.52i.).

### Total RNA extraction and quantitative reverse transcription-polymerase chain reaction (qRT-PCR)

Cells were harvested in TRIzol reagent (Sigma-Aldrich, T924) before the addition of 1-bromo-3-chloropropane (Sigma-Aldrich, B9673). After centrifugation (15300xg, 15 min, 4° C) the aqueous phase was collected, incubated for 10 min in propan-2-ol (VWR, 20842.298) before centrifugation (15300xg, 10 min, 4° C). After removing the supernatant, the pellets, containing RNA, were washed twice with 75% ethanol and dissolved in sterile water by heating for 10 min at 50° C. RNA were quantified by using a NanoDrop 2000 Spectrophotometer (Thermo Fisher Scientific, USA). Reverse transcription was performed using the RevertAid First Strand cDNA Synthesis Kit in accordance with the supplier’s instructions (Thermo Fisher Scientific, K1632). qPCR was then performed in LightCycler 480 (Roche Applied Science, Penzberg, Germany) by mixing 10 ng of cDNA with the SYBR Green PCR Master Mix (Promega, A6002, USA) and 0.5 μM of forward and reverse primers further described in Table 1. The template was initially denatured for 5 min at 95° C, followed by 40 amplification cycles of 10 sec at 95° C, 15 sec at annealing temperature and 10 sec at 72° C. Results were analyzed with LightCycler480 software (Roche Applied Science, version SW 1.5). The expression level of a target genes was normalized relative to that of 36b4.

**Table 1:** Primers used for qRT-PCR amplification.

**Gene**	**Sense**	**Sequence (5’-3’)**	**Amplicon size**	**Annealing temperature**
*36b4*	Forward	AGC TGA AGC AAA GGA AGA GTC GGA	84bp	58° C
	Reverse	ACT TGG TTG CTT TGG CGG GAT TAG		
*Mcp-1*	Forward	TCA CCT GCT GCT ACT CAT TCA CCA	98bp	58° C
	Reverse	TAC AGC TTC TTT GGG ACA CCT GCT		
*IL-6*	Forward	ATC CAG TTG CCT TCT TGG GAC TGA	134bp	58° C
	Reverse	TAA GCC TCC GAC TTG TGA AGT GGT		
*IL-1β*	Forward	AAG GGC TGC TTC CAA ACC TTT GAC	100bp	57° C
	Reverse	ATA CTG CCT GCC TGA AGC TCT TGT		
*Tnf-a*	Forward	TCT CAT GCA CCA CCA TCA AGG ACT	92bp	58° C
	Reverse	ACC ACT CTC CCT TTG CAG AAC TCA		
*P16^INK4A^*	Forward	GAA CTC TTT CGG TCG TAC CC	85bp	57° C
	Reverse	ATC TGC ACC GTA GTT GAG C		
*Tp53*	Forward	CGT AAA CGC TTC GAG ATG TTC C	140bp	58° C
	Reverse	TTA TGG CGG GAA GTA GAC TGG		
*P21^CIP1^*	Forward	ACG GTC GAA CTT TGA CTT CG	148bp	
	Reverse	AGT ACT GGG CCT CCT GTC C		55° C

### Western blot

Cells were harvested on ice with RIPA lysis buffer (Sigma-Aldrich, RO278) containing Halt™ Protease Inhibitor Cocktail (Thermo Fisher Scientific, 78429) and Halt™ Phosphatase Inhibitor Single-Use Cocktail (Thermo Fisher Scientific 78420). The samples were centrifugated (15300xg, 15 min, 4° C) and supernatants were collected. Protein concentration was determined using the Pierce™ Bicinchoninic Acid Protein Assay Kit (Thermo Fisher Scientific, 23225) according to the supplier’s instructions. Protein samples (30 μg) were diluted in Laemmli buffer solution (Bio-Rad, 161-0747, USA) containing 2-mercaptoethanol (Bio-Rad, 161-0710) (dilution ratio 4:1). Samples were heated 6 min at 95° C then loaded and separated by SDS-PAGE using 4-15% polyacrylamide gel, and transferred to a 0.45 μm nitrocellulose membrane (Bio-Rad, 1620115). Membranes were blocked with Tris buffered saline (Thermo Fisher Scientific, 28358) containing Tween 0.2% (v/v) and non-fat milk 5% (w/v) for 1h at room temperature (RT). Primary antibodies were incubated overnight in TBS-Tween 0.2%, 5% milk at 4° C as follows: p16^INK4A^ (1/500, Sigma-Aldrich, ZRB1437), p21^CIP1^ (1/750, Abcam, ab188224, UK), p53 (1/400, R&D Systems, AF1355, USA) and β-Actin (1/5000, Sigma-Aldrich, A5441). Membranes were washed with TBS-Tween 0.2% and horseradish peroxidase conjugated secondary antibodies (1/5000, Sigma-Aldrich, A6154) were incubated 1h at RT further washed as previously described. Chemiluminescence was determined using the ECL detection system (Bio-Rad, 170-50600). Images were acquired using a Chemidoc MP imager (Bio-Rad). Signal intensities were quantified by densitometry using ImageJ software (NIH, v1.52i.).

### Multiplex analysis

The SASP components were analyzed using the Meso Scale Discovery (MSD®) assay kits. The method was carried out following the manufacturer’s instructions (U-PLEX custom biomarker group 1, cat N° K15069M-1). For this experiment, 1mL of macrophage conditioned media was collected at day 2, 7 and 14 after 24h FBS deprivation. For the detection of Interleukin-6 (IL-6), Keratinocyte Chemoattractant-Growth Regulated Oncogen (KC-GRO), Monocyte Chemoattractant Protein-1 (MCP-1), Matrix-Metalloproteinase-9 (MMP-9) and Tumor Necrosis Factor alpha (TNF-α), the conditioned media were diluted (1:1) with the diluent assay. However, to detect interleukins IL-1β and IL-10, the conditioned media were used without any dilution. Protein levels were determined using a MESO QuickPlex SQ120 (MSD, USA) and calculated using a standard curve with software provided by the manufacturer (MSD Discovery Workbench).

### SeaHorse metabolic analysis

Oxygen consumption rate (OCR) and extracellular acidification rate (ECAR) were analyzed on a Seahorse XFe24 extracellular flux analyzer (Agilent Technologies) according to the manufacturer’s instructions. Briefly, cells were washed twice with XF RPMI medium pH 7.4 (Agilent Technologies, 103576-100) containing either 10mM glucose (Thermo Fisher Scientific, A24940-01), 2mM glutamine (Thermo Fisher Scientific, 25030024) and 2mM sodium pyruvate (Thermo Fisher Scientific, 11360-070) for OCR measurements, or supplemented only with 2mM glutamine (Thermo Fisher Scientific, 25030024) for ECAR measurements. Cells were then incubated with the same medium for 1 h in a CO_2_-free incubator at 37° C to allow temperature and pH equilibration. OCR was measured in basal rates with 3 measurement cycles and after sequential injections of 1 μM oligomycin (Sigma-Aldrich, 75351), 1 μM Carbonyl Cyanide 4-(trifluoromethoxy) phenylhydrazone (FCCP) (Sigma-Aldrich, SML2959) and a 0.5 μM mix of rotenone/antimycin A (Sigma-Aldrich, R8875 and A8674 respectively) with 3 measurement cycles after each injection and 3 final measurement cycles. ECAR was measured in basal rates with 3 measurement cycles and after sequential injections 10 mM glucose (Sigma-Aldrich, G-8270), 1 μm oligomycin (Sigma-Aldrich, 75351) and 20 mM 2-deoxy-D-glucose (Sigma-Aldrich, D8375) with 3 measurement cycles after each injection and 3 final measurement cycles. At the end of the experiment, the cells were fixed for 8 min with 10% Formalin (Sigma-Aldrich, HT501128) and then the nuclei were stained 10min with Hoescht 33342 (1/2000, Invitrogen, H3570). Images were acquired using a THUNDER fluorescence microscope (Leica, Wetzlar, Germany). The nuclei were then counted using ImageJ software (NIH, v1.52i.). OCR and ECAR values were then normalized to cell number in order to compare the different conditions.

### Proliferation assay

Cultured macrophages were treated with 10 μM 5-Ethynyl-2′-deoxyuridine (EdU) for 24h, fixed 8 min with 10% Formalin (Sigma-Aldrich, HT501128) and then permeabilized 15 min with PBS-Triton 0.2% (v/v). EdU incorporation was detected using Click-iT EdU Cell Proliferation (Invitrogen, C10337) according to the supplier’s instructions. Then nuclei were stained 10 min with Hoescht 33342 (1/2000, Invitrogen, H3570). Fluorescence was detected using a THUNDER fluorescence microscope (Leica, Germany). EdU positive cells were quantified using ImageJ software (NIH, v1.52i.).

### Terminal deoxynucleotidyl transferase dUTP nick-end labeling (TUNEL) assay

Cells were detected using the *In Situ* Cell Death Detection Kit, Fluorescein (Roche, 11684795910) according to the manufacturer’s instructions. Briefly, cells were fixed for 10 min with 10% Formalin (Sigma-Aldrich, HT501128) and permeabilized for 15min with 0.2% (v/v) PBS-Triton. As a positive control, cells were incubated 20 min at RT with 5U/μL of deoxyribonuclease-I (DNAse I) (Promega, Z358A) in a humidified atmosphere. Apoptotic cells were then labeled with the TUNEL reaction mixture for 1 h at 37° C and 5% CO_2_ in a humidified atmosphere out of the light. Cells were washed twice with PBS and nuclei were stained 10 min with Hoescht 33342 (1/2000, Invitrogen, H3570). Fluorescence was detected using a THUNDER fluorescence microscope (Leica, Germany). Apoptotic positive cells were quantified using ImageJ software (NIH, v1.52i.).

### Phagocytosis assay

Phagocytosis activity was quantified using Molecular Probes™ pHrodo™ Red Zymosan Bioparticles™ Conjugate (Invitrogen, P35364). Zymosan BioParticles are resuspended in sterile PBS (0.5 mg/mL) and homogenized 10 min at 40kHz in a sonication bath (Emerson, USA). Cultured macrophages were incubated for 3h at 37° C, 5% CO_2_ with Zymosan BioParticles: 60 μg/millions of cells. Then, cells were washed with PBS and fixed for 5 min with 10% Formalin (Sigma-Aldrich, HT501128). Cells were dissociated by incubation for 10 min at 4° C with Macrophage Detachment Solution (PromoCell, C-41330, Heidelberg, Germany) and peeled off by gentle scraping before centrifugation (500xg, 5min, RT) to remove Macrophage Detachment Solution. The pellet was resuspended in PBS and cell fluorescence was quantified and analyzed by flow cytometry using MACSQuant VYB (Miltenyi Biotec, Westphalia, Germany) and FlowJo software (BD Biosciences, version 10.8.1, USA) respectively.

### Senolytic and cell apoptosis assay using flow cytometry

Cells were seeded at 1.10^6^ cells per well (12 well plate). From seeding, macrophages were treated or not at D2, D7 or D14 with dasatinib (TargetMol, T1448, USA) and quercetin (TargetMol, T2174, USA) for 24h. Culture supernatant was collected and cells were washed with PBS before dissociation using trypsin 0.25% and EDTA 0.2 g/L (Sigma-Aldrich, T4049). Dissociation was stopped adding serum-containing culture supernatant. Cells were peeled off by gentle scraping before centrifugation (110xg, 5min, 4° C) and the pellet was resuspended in RPMI 1640 medium with Glutamax™ containing FBS and antibiotics as previously described (nearly 2.10^6^ cells/mL). Apoptosis and cell death assay were carried out using Muse Annexin V & Dead Cell kit (Cytek Biosciences B.V., MCH100105, Amsterdam, The Netherlands). Resuspended cells were mixed with the Muse Annexin V and Dead cell reagent according to the manufacturer instructions. The percentage of apoptotic cells was analyzed by flow cytometry using a Muse cell analyzer (Cytek Biosciences B.V., Amsterdam, The Netherlands).

### Statistical analysis

All data are presented as the mean ± SEM from at least four or more biological replicates. The differences between data sets were assessed by a non-parametric t-test (Mann-Whitney) to compare the effect of CB3 between groups or a non-parametric one-way ANOVA (Kruskal-Wallis) with Dunn multiple comparison test to analyze groups overtime using GraphPad Prism (GraphPad Software, version 10). Statistical significance levels are indicated in the figures using asterisks as follows: # *p* < 0.05, ## *p* < 0.01, ### *p* < 0.001, * *p* < 0.05, ** *p* < 0.01, *** *p* < 0.001, and **** *p* < 0.0001.

## Supplementary Material

Supplementary Figures
