# Supplementary material for: A new model and precious tool to study molecular mechanisms of macrophage aging
Source: Aging (Albany NY). 2024 Oct 3;16(19):12697–725. doi: 10.18632/aging.206124 (PMC11501386; doi:10.18632/aging.206124)
Supplement: Supplementary Figures [file aging-16-206124-s001.pdf]

SUPPLEMENTARY FIGURES

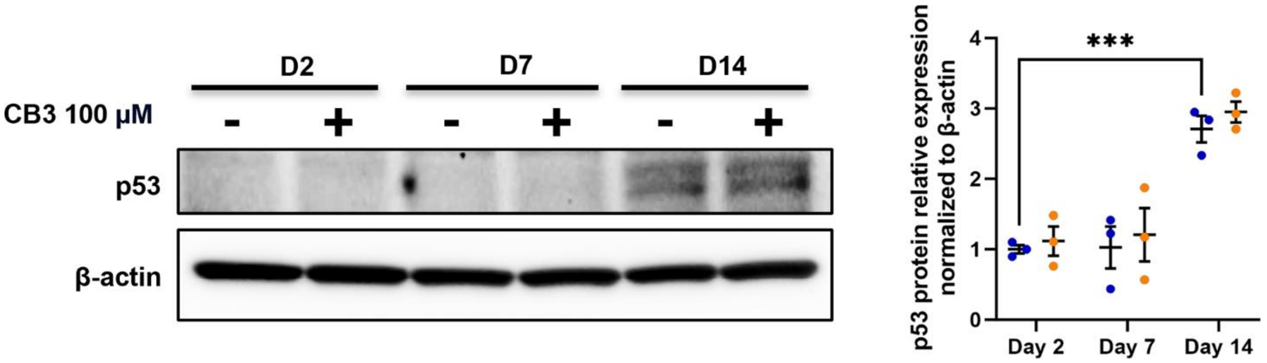

Supplementary Figure 1. p53 analysis in aged macrophages *in vitro*. Murine peritoneal macrophages from young mice (3 months) were cultured during 2, 7 or 14 days *in vitro*. They are chronically treated with 100 µM CB3 (yellow) for up to 14 days or not (blue). Left panel: immunoblot for p53. Right panel: quantification by densitometric analysis (n=3). Error bars represent the mean ± SEM. p-values were obtained comparing groups overtime using a non-parametric one-way ANOVA analysis (Kruskal-Wallis analysis followed by Dunnett's multiple comparison test; ****p*<0.001).

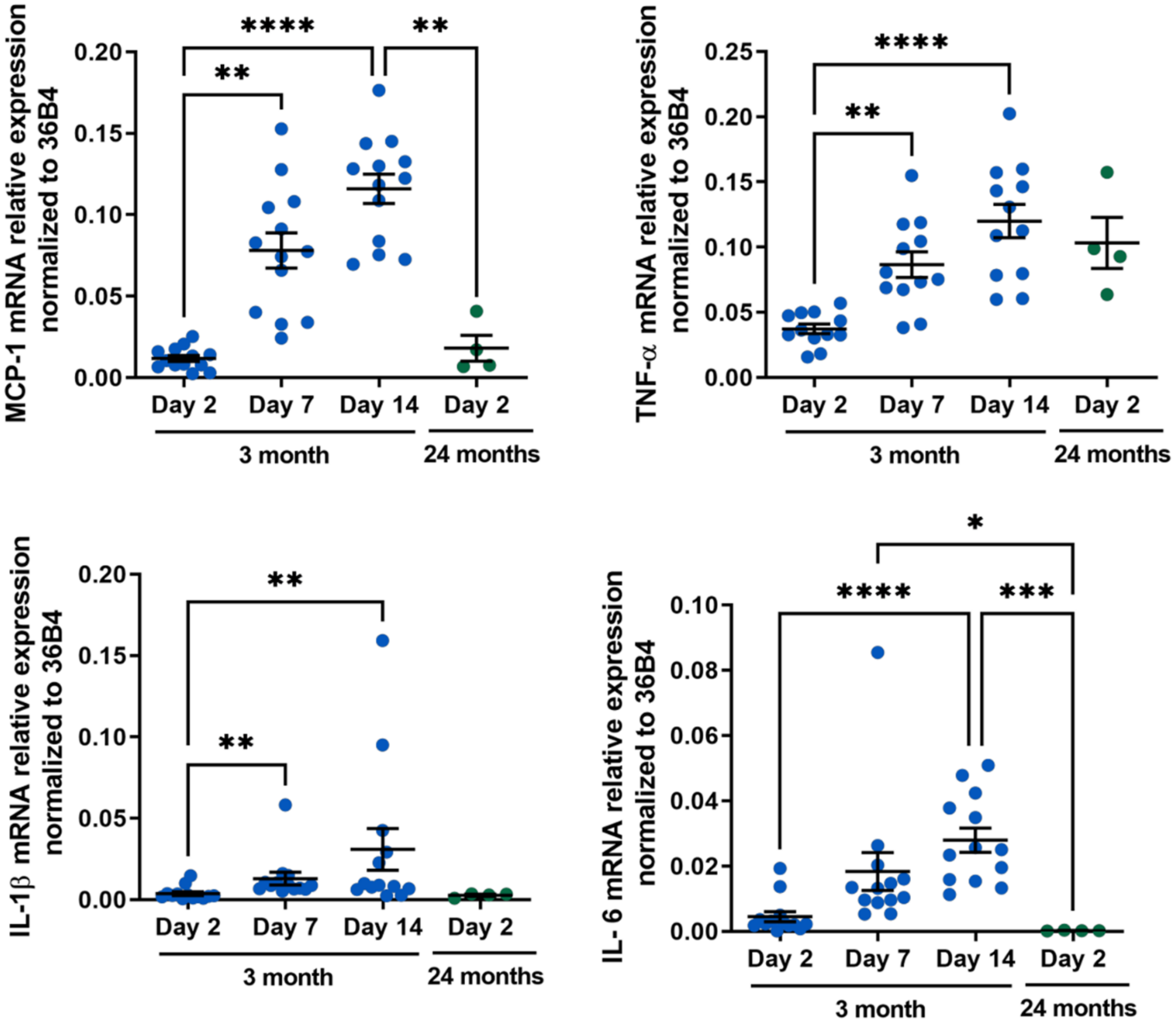

Supplementary Figure 2. SASP analysis in aged macrophages *in vitro*. Murine peritoneal macrophages from young mice (3 months) were cultured during 2, 7 or 14 days *in vitro*. All results are compared to murine peritoneal macrophages from 24 months old mice cultured for 2 days. RT-qPCR analysis for SASP markers MCP-1, IL-1b, TNF-a and IL-6 transcripts normalized to 36B4 (n=13). Error bars represent the mean ± SEM. p-values were obtained comparing groups overtime using a non-parametric one-way ANOVA analysis (Kruskal-Wallis analysis followed by Dunnett's multiple comparison test; $*p<0.05$; $**p<0.01$; $***p<0.001$; $****p<0.0001$).

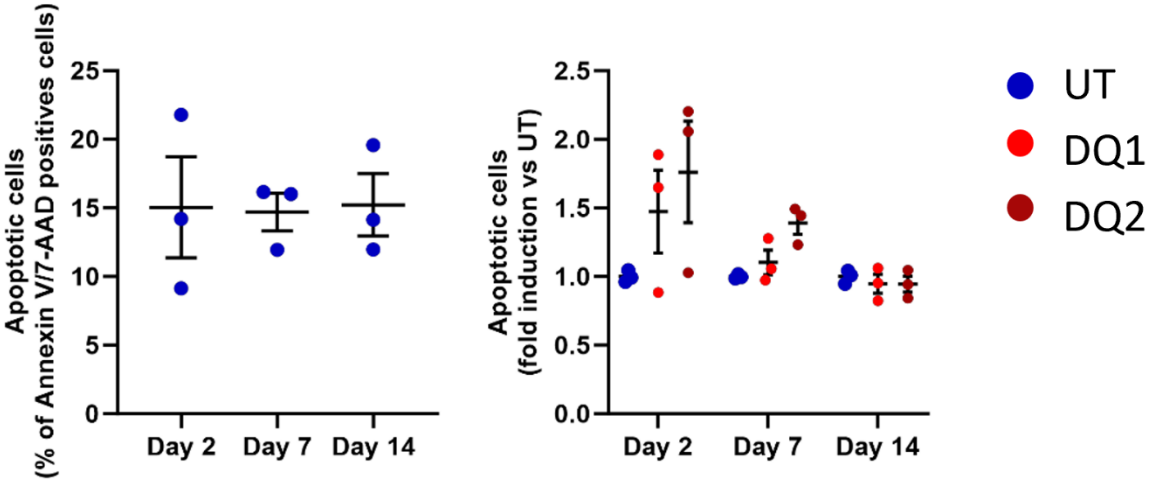

Supplementary Figure 3. Effects of senolytic drugs on aged macrophages *in vitro*. Murine peritoneal macrophages from young mice (3 months) were cultured during 2, 7 or 14 days *in vitro* and treated or not (UT) with Dasatinib 250 nM and quercetin at 15 µM (DQ1) or 30 µM (DQ2) for 24h. Apoptosis and cell death have been quantified by flux cytometry using annexin V and 7-AAD labeling (left panel; n=3). % of Annexin V and 7-AAD positive cells compared to untreated cells was also quantified (right panel). Error bars represent the mean ± SEM. p-values were obtained comparing groups overtime using a non-parametric one-way ANOVA analysis (Kruskal-Wallis analysis followed by Dunnett's multiple comparison test).

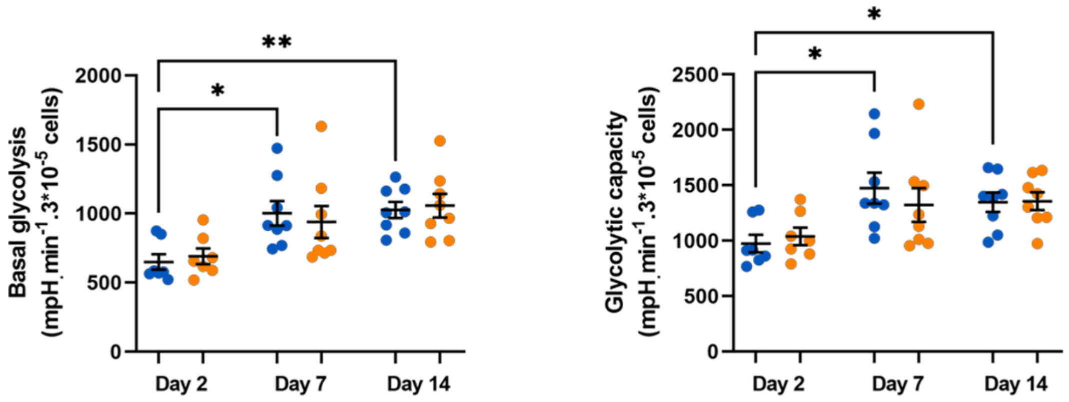

Supplementary Figure 4. CB3 effects on metabolic phenotype in aged macrophages *in vitro*. Macrophages are chronically treated with 100uM CB3 (yellow) or not (blue) for up to 14 days. Left: analysis of basal glycolysis. Results are normalized by cell count using DAPI staining (n=3). Right: analysis of glycolytic capacity. Results are normalized by cell count using DAPI staining (n=8). Error bars represent the mean ± SEM. p-values were obtained comparing groups overtime using a non-parametric one-way ANOVA analysis (Kruskal-Wallis analysis followed by Dunnett's multiple comparison test; *$p<0.05$; **$p<0.01$).

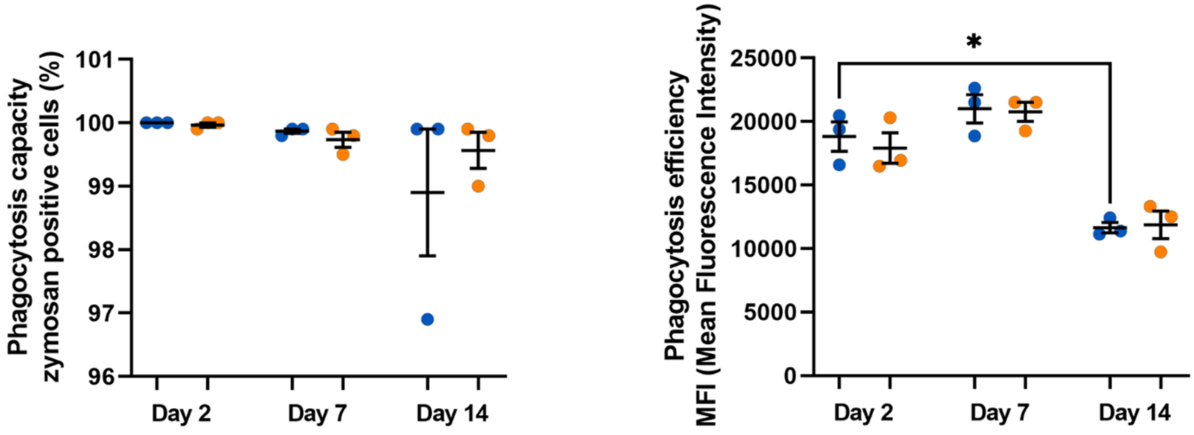

Supplementary Figure 5. CB3 effects on phagocytosis in aged macrophages *in vitro*. Macrophages are chronically treated with 100 uM CB3 (yellow) or not (blue) for up to 14 days. The ability of aged macrophages to phagocytose was analyzed after 3h of pHrodo Red zymosan treatment. Left panel: quantification of the number of phagocytic macrophages (% of positive cells). Right panel: quantification of the mean fluorescence intensity (MFI) representing phagocytosis efficiency (n=3). Error bars represent the mean ± SEM. p-values were obtained comparing groups overtime using a non-parametric one-way ANOVA analysis (Kruskal-Wallis analysis followed by Dunnett's multiple comparison test; *$p<0.05$).
